# Branchial Pathomorphology of Southern Bluefin Tuna *Thunnus maccoyii* (Castelnau, 1872) Infected by Helminth and Copepodan Parasites

**DOI:** 10.3389/fphys.2017.00187

**Published:** 2017-03-29

**Authors:** Mark B. Adams, Craig J. Hayward, Barbara F. Nowak

**Affiliations:** Department of Fisheries and Aquaculture, Institute for Marine and Antarctic Studies, University of TasmaniaLaunceston, TAS, Australia

**Keywords:** tuna, parasite, gills, histopathology, helminth, copepod, leucocyte

## Abstract

Three metazoan parasites, a monogenean *Hexostoma thynni* and two species of copepods *Pseudocycnus appendiculatus* and *Euryphorus brachypterus* are known to parasitize the gills of ranched southern bluefin tuna (SBT) and other tuna species. However, there is no detailed information describing the pathological response to infection by these parasites in this species. Wild southern bluefin tuna *Thunnus maccoyii* (approximately 3 years of age), captured and towed to a grow-out site in the waters immediately south of Port Lincoln, South Australia were subsequently sampled (*n* = 10) monthly from March until August 2004 during commercial harvest operations. Longitudinal sections of gill hemibranchs with attached parasites were excised and fixed for routine histology and immunohistochemistry. Reference samples were also collected from fish displaying no signs of parasitism or other grossly observable anomalies. Two morphologically distinct granulocytes were observed and putatively identified as eosinophils and mast cells. Pathology was localized to filaments upon and immediately adjacent to parasite attachment sites. Branchial cellular responses, adjunct to the attachment of *H. thynni* by its opisthaptoral clamps, included hyperplasia and inflammation resulting in structural remodeling of branchial tissues. Inflammatory infiltrates were often dominated by putative eosinophils and lymphocytes when parasitized by *H. thynni* and *P. appendiculatus*. Gill associated lymphoid tissue infiltrated the lamellar regions particularly in response to helminth infection. A variable response ranging from hemorrhage with minor hyperplasia or fibroplasia and eosinophilic inflammation to a barely discernible change was seen for gill sections harboring *P. appendiculatus* and *E. brachypterus*. The magnitude of the host response to attachment by the latter was congruent with attachment proximity and parasite load. On the basis of the host responses reported here and the low intensity of infection observed in other associated studies these gill ectoparasites are currently considered a low risk for wild and ranched adult SBT.

## Introduction

Metazoan fish parasites include an extremely numerous and diverse array of organisms (for review see Rohde, 2005). The phyla Platyhelminthes and Arthropoda contribute significantly to the overall volume of metazoan parasites hosted by fish. These phyla contain a vast number of parasitic flatworms and copepods able to attach, feed and reproduce upon the gills and skin of various fish species (Hayward, 2005; Makepeace et al., 2012). Although parasitism of fish is generally considered a benign activity amongst wild fish populations, under culture conditions the opportunity for parasites to rapidly proliferate is potentially enhanced (Kent, 2000; Nowak, 2007). Ranching of southern bluefin tuna *Thunnus maccoyii* (Castelnau, 1872) (SBT) is one of the biggest fish production industries in Australia. Ranching was developed by the SBT industry during the early 1990s as a result of reduction in the total allowable catch. The wild fish (2–4 years old) are caught in the Great Australian Bight and towed to ranching areas in the Spencer Gulf near Port Lincoln where they are fattened over 3–6 months and then sold mostly on the Japanese market.

While blood flukes have been the main health problem for the SBT industry (Colquitt et al., 2001; Aiken et al., 2006; Hayward et al., 2009; Kirchhoff et al., 2011; Hardy-Smith et al., 2012; Polinski et al., 2013), gill ectoparasites, including one species of monogenean *Hexostoma thynni* (Delaroche, 1811) and two species of copepods *Pseudocycnus appendiculatus* (Heller, 1865) and *Euryphorus brachypterus* (Gerstaecker, 1853) have been commonly reported (Deveney et al., 2005; Hayward et al., 2007, 2008; Kirchhoff et al., 2011). These gill parasites have been found on other species of tuna (Nowak et al., 2006; Aiken et al., 2007; Mele et al., 2010, 2012, 2015; Culurgioni et al., 2014; Pleić et al., 2015) but their impact on SBT has not been investigated. While these infections are usually not an issue, it is possible that they could become problematic if there are any changes in the infection intensity of the wild stock or where tuna production is based on hatchery-raised juveniles.

*Hexostoma thynni* is a polyopisthocotylean monogenean. This group of monogeneans feed on host blood and some species, for example *Zeuxapta seriolae* (Meserve, 1938), have been shown to cause anemia (Mansell et al., 2005). Polyopisthocotyleans use opisthaptoral clamps to attach to gill lamellae (Hayward, 2005). Compared to their monopisthocotylean counterparts, significant gill damage is somewhat limited in scope (Hayward, 2005; Wootten, 2012) possibly to facilitate a sustained, unimpeded blood draw from the underlying vasculature. However, for fish species parasitized by polyopisthocotyleans, swollen filaments (sub-gross) presumably comprised of lamellae fused with hyperplastic epithelium commonly manifests. Other pathologies in concert with hyperplastic changes may include inflammation, hemorrhage, occluded fibrin, and necrosis (Montero et al., 2004; Mansell et al., 2005; Rubio-Godoy, 2007; Clarke et al., 2012). The degree of pathological change ranges from discrete to occasionally severe (Rubio-Godoy, 2007) which is possibly dependent upon the parasites feeding activity, opisthaptor size and complexity, duration of infection, and the size/age of host. Infection of tuna (Linnaeus, 1758) with *H. thynni* (under the name *Neohexostoma extensicaudum* Price, 1961) resulted in swelling of the gill around attachment sites, hyperplasia, and erosion of gill epithelium (Dawes, 1940). However, detailed information describing the cellular host response of SBT to infection with *H. thynni* is lacking.

*P. appendiculatus* is a siphonostomatoid copepod also found to parasitize other tuna families. Haemorrhage, necrosis, apoptosis, mucosal hyperplasia were localized changes described for Atlantic tuna *Thunnus thynnus* (Linnaeus, 1758) gills with *P. appendiculatus* (Pleić et al., 2015). Another related species from this family of copepods, *Cybicola armatus* (Bassett-Smith, 1998) (cited under the name *P. armatus*) caused significant gill changes including tissue erosion and inflammation in its scombrid host *Scomberomorus guttatus* (Bloch and Schneider, 1801; Fowler, 1905; cited under the name *Indocybium guttatum*), particularly in the area of attachment, and an increased number of mucous cells in affected filaments (Natarajan and Nair, 1972). *Euryphorus brachypterus*, another copepod from the order Siphonostomatoida caused gross damage by their attachment to the host, including hemorrhage (Williams and Bunkley-Williams, 1996). However, there is little information on the effects of *P. appendiculatus* and *E. brachypterus* on SBT at the cellular level.

Given the host response of southern bluefin tuna (SBT) gills from a pathomorphological perspective is largely unexplored, the aim of this study was to describe the pathological changes caused by the metazoan ectoparasites *H. thynni, P. appendiculatus*, and *E. brachypterus* commonly found upon the gills of ranched SBT.

## Materials and methods

### Fish collection, sampling, and tissue preparation

Tissue samples were collected on an opportunistic basis from fish being killed during commercial fishing and harvest operations. The fish were killed using commercial harvesting techniques and tissues collected post mortem as follows. Fish were killed by iki-jime, bled by pectoral cuts, cored with a “tanguchi tool,” brain and upper spinal nerves destroyed by wiring and finally the gills and viscera were excised and transferred to plastic bags immersed in a slurry of ice. Longitudinal sections of gill hemibranchs with attached parasites were excised and fixed in 10% formalin within 5–10 min of death.

The collection, transfer, holding, and initial sample collection of SBT *Thunnus maccoyii* analyzed herein are as per methods described by Hayward et al. (2007) and Kirchhoff et al. (2011). Briefly, wild SBT *Thunnus maccoyii* (approximately 3 years of age), were caught using a purse seine net in the fishing grounds of the Great Australian Bight (map reference 132.21, 33.3). After capture the tuna were towed to a grow-out site in the waters immediately south of Port Lincoln, South Australia and transferred into sea cages for a commercial on-growing of ~6 months. The gills of 10 tuna from this cohort were collected from fish caught by baited hook and line prior to transfer of the fish into sea-cages 3 weeks later. Further gill samples were collected over the pursuant 6 months on a monthly basis (*n* = 70) in concert with opportunistic collections of tuna parasitized by *Euryphorus* sp. during 2010 (*n* = 8). Tuna were hand-captured by a diver (on snorkel) targeting a small part of the school within a cage that had been segregated and condensed by seine net.

### Histochemistry and immunohistochemistry

**Figure 1:**
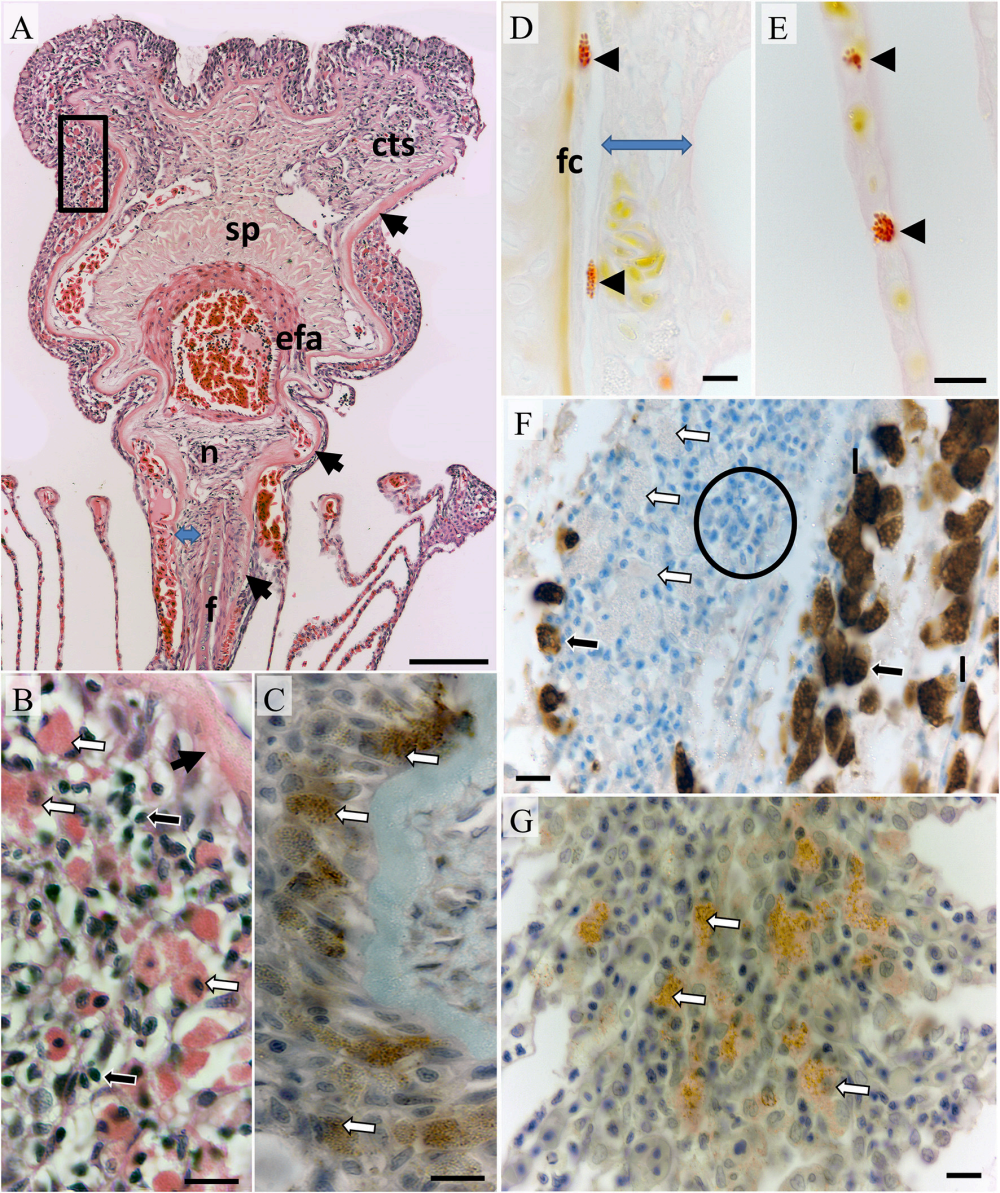
**Morphology of an un-parasitized gill filament. (A)** The leading third of a filament sectioned in the dorsal plane showing the filament core (f), surrounding connective tissue region (blue arrow), basal lamina (black arrows) encapsulating connective tissue, nerve bundle (n) and vascular components including the efferent artery (efa), spongiosa (sp), and connective tissue with prominent sinusoids and collateral vessels (cts); Bar = 100 μm (H&E). **(B)** Higher magnification of boxed region above showing intraepithelial putative mast cells (white arrows) and small mononuclear cells (black arrows); Bar = 100 μm (H&E). **(C)** DAB+ve immuno-stained cells of similar size and granularity in a serial section from the same tissue as previous, basal lamina (unbordered black arrow); Bar = 10 μm (mouse anti-human tryptase diluted 1:100). **(D)** Elongated Bierbrich scarlet positive granulocytes within connective tissue embedded vessels located next to the filament core and underlying the filamental epithelium (blue arrow) with a rounded form circulating through lamellar pillar channels in plate **(E)**; Bars = 10 μm. **(F)** Chloride cells DAB+ve for Na/K/ATPase (black arrows) in the trailing filamental region located upon lamellae (l) while putative mast cells are unlabeled (white arrows). Filamental epithelium was heavily infiltrated with small mononuclear cells in the trailing region typical of a lymphoid parenchyma (example indicated within circle); Bar = 10 μm. **(G)** DAB+ve immuno-stained mast cells within the lamellar juncture; Bar = 10 μm (mouse anti-human tryptase diluted 1:100). Sections were cut serially in the dorsal plane except **(D,E,G)** which were realigned in the saggital plane.

Following fixation (24 h) the gill samples were further trimmed to permit placement into tissue cassettes and processed for routine histology. Sections (5 μm) were cut along the dorso-ventral hemibranch axis at depths of approximately 100, 500, and 1,000 μm (Figure 1A). Where additional samples from each fish were available, transverse sections in the dorsal plane were cut through the excised filaments. Following dehydration, serial sections were stained with haematoxylin & eosin (H&E). Initial observations of H&E stained gill sections revealed three morphologically distinct eosinophilic cell morphotypes of which two featured prominent granules. To demarcate these morphotypes a selection of representative samples (*n* = 8; two for each parasitized and non-paraisitized fish) were serially sectioned then profiled by histochemistry and immunohistochemistry (IHC). Sections were stained with periodic acid/Schiff's reagent both alone and in combination with Alcian blue pH 2.5 (mucus cells) or Biebrich scarlet and metanil yellow (eosinophils) (Tomasi et al., 2008).

Sections for tryptase IHC were mounted on polysine slides (Thermo Fisher Scientific), hydrated and placed in a proprietary antigen retrieval solution (DAKO S170084-2), and autoclaved (30 min). Sections were transferred after cooling to buffer (PBS + 1% BSA) then endogenous peroxidase activity was blocked (DAKO Envision System kit, K4006). Sections were rinsed in dH_2_O, immersed in buffer and incubated (30 min, room temp, 1:100) with mouse anti-human mast cell tryptase (DAKO M7052), which cross reacts with perciforme mast cells (Balla et al., 2010; Balci et al., 2014). Zebrafish gills served as a tissue control and a universal negative control (DAKO IR75066) was used for isotype control. Sections were then incubated with anti-mouse HRP polymer labeled secondary antibody (30 min, room temp), rinsed in buffer, and flooded with diaminobenzidine (DAB) solution (5 min) (Envision System HRP, cat. no. K4006; DAKO).

Sections for Na^+^/K^+^-ATPase IHC were mounted on Vectabond™ (Vector Laboratories, Burlingame, CA, USA) coated slides, hydrated, immersed in citrate buffer solution (pH 6), micro-waved on high for 12 min, cooled, rinsed (dH_2_O), and blocked for endogenous peroxidase with 3% H_2_O_2_ (20 min). Following a rinse with PBS (3 × 1 min) and incubation with normal horse serum (Vector Laboratories) (20 min) the sections were then blotted dry and incubated in a humid chamber at 37.5°C (60 min) with mouse anti-avian Na+/K+-ATPase (1:100, IgGα5, Developmental Studies Hybridoma Bank, University of Iowa) which labels chloride cells from a range of teleost families (MacDonald et al., 2002; Adams and Nowak, 2003; Anthony et al., 2007; Shin et al., 2009). Gills from Atlantic salmon served as a tissue control and normal mouse serum was used for isotype control. Sections were rinsed, incubated at 37.5°C (30 min) with biotinylated horse anti-mouse IgG (ABC kit; Vector Laboratories) (1:200), rinsed and incubated (30 min, room temp) with peroxidase conjugated streptavidin (1:200 ABC kit: Vector Laboratories). After a final washing step, the slides were flooded with 3,3′-diaminobenzidine (DAB) in peroxide buffer (2 min) (Roche Diagnostics) then rinsed in deionized water for 30 s before counterstaining with Mayer's haematoxylin for (30 s), then rinsed, differentiated in PBS for 30 s, dehydrated, cleared, and mounted. All slides were counterstained with Mayer's hematoxylin, blued in PBS, dehydrated and mounted.

Slides were examined using an Nikon Ni-U compound light microscope and images digitally captured from a Nikon DS-Ri2 microscope camera interfaced with NIS-Elements BR imaging software.

## Results

### Morphology of non-parasitized gill tissues

The core of each filament consisted of bone, cartilage, and chondroid tissue overlaid with connective tissue (Figure 1A). Although commencing as a bulbous construct in the trailing edge of the filament the core is mostly blade-like terminating below a prominent nerve bundle underlying the efferent artery (Figure 1A). The connective tissue layer thickens toward the filament peripheries and is interlaced with sinusoids. The basal lamina of the filamental epithelium overlays connective tissue constituents simultaneously extending and thickening to encapsulate the nerve bundle, efferent artery and collateral vessels (Figure 1A). The efferent artery has a prominent associated spongiosa capped with sinusoidal connective tissue (Figure 1A). Occasional leukocytes including elongated granulocytes containing large granules positive for Biebrich scarlet, negative for tryptase, and variably PAS positive were found in the connective tissue regions (Figure 1D). Rounded forms with identical staining properties and also containing large granules and peripheral nuclei were occasionally observed within larger sinusoids, vessels, and lamellae blood channels (Figure 1E; referred to putatively as eosinophils here on).

Overlaying the basal laminar and underlying the mucosa intraepithelial leucocytes and large finely granular eosinophilic cells whose granules bound tryptase antibody but not Biebrich scarlet or PAS (referred to putatively as mast cells here on; Figures 1B,C). A similar arrangement was observed toward the trailing edge of filaments and at the base of filaments embedded in the interbranchial septum although lymphocytes featured more prominently (Figure 1F). Chloride cells with typically foamy eosinophilic cytoplasm were positive for Na^+^/K^+^-ATPase. This antibody was not bound by eosinophils or mast cells located sub-mucosal regions or in connective tissues (Figure 1F). Chloride cells were common in the trailing edge filamental mucosa and extending along the epithelium of lamellae. The anterior third of each lamella on adjacent filaments was embedded distally within an epithelial juncture. Mast cells were common underlying the juncture's leading edge mucosa (Figure 1G) and were morphologically similar to those found in filamental epithelia.

### Pathomorphology of parasitized gill tissues

#### Hexastoma thynni

**Figure 2:**
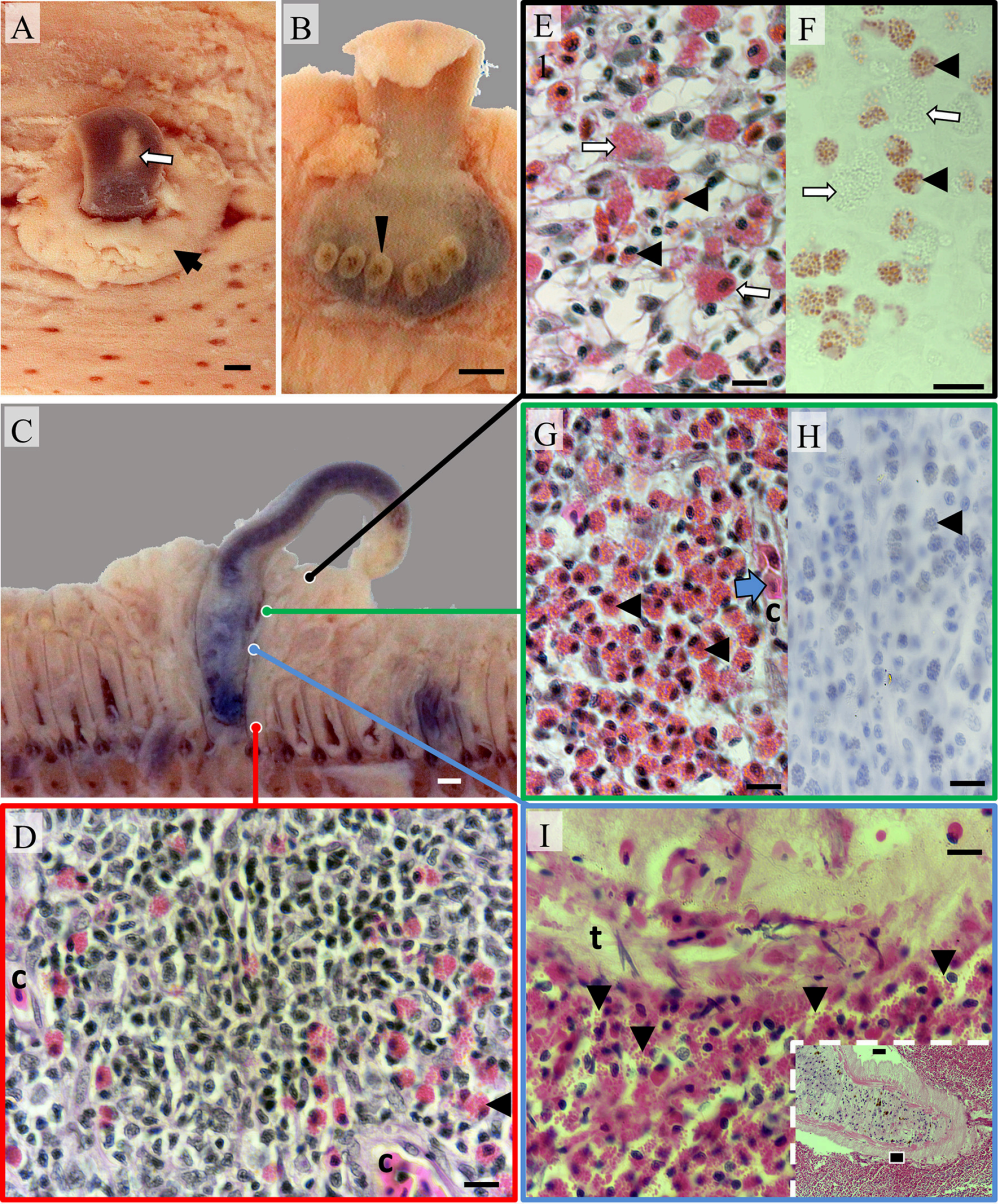
**Pathomorphology of gill filaments parasitized by ***Hexastoma thynni***. (A)** The leading edge of a fixed hemibranch showing a fluke (white arrow) protruding from a mass of swollen tissue (black arrow); Bar = 1 mm. **(B)** The opisthaptor of an embedded fluke with six haptoral clamps displayed (arrowhead) **(C)** A cross sectioned fluke and surrounding tissue—note substantial distortion of adjacent filaments; Bar = 1 mm. **(D)** Typical tissue morphology encountered in the trailing filament region where small intrapithelial mononuclear cells featured prominently. The tissue was vascularized with capillaries (c); Bar = 10 μm (H&E). **(E)** Eosinophils (black arrowheads) infiltrated the spongiotic epithelial stroma in the leading edge with a variable presence of mast cells (white arrows); Bar = 10 μm (H&E). **(F)** Serial section showing eosinophils positive for Bierbrich scarlet (black arrowheads) and mast cells negative for the stain (white arrows); Bar = 10 μm. **(G)** Medial regions of swollen tissue were heavily infiltrated with eosinophils (black arrowheads) with erythrocytes (blue arrow) commonly found within capillaries (c). Eosinophils were negative for tryptase (shown in **H**—black arrowheads); Bar = 10 μm. **(I)** Inset showing a fluke surrounded by eosinophilic cells (bar = 1 mm). The black box indicates the position of the image taken in the main plate where degranulating eosinophils (arrowheads) were present in large numbers at the host-pathogen interface and adhered to the tegument of *H. thynni* (t); Bar = 10 μm (H&E).

Upon excision of gills occasional attached flukes were observed to be contracted, non-motile, and pale in appearance. An opaque mucoid like layer, removable by forceps, was also detected on some individuals. A pronounced focal swelling of gill tissues surrounded flukes embedded between adjacent filaments (Figure 2A). The majority of flukes were located proximally to the arch and were secured by six opisthaptor clamps whilst the prohaptor protruded beyond the leading edge of the filaments (Figures 2B,C). Occasionally these characteristic lesions were devoid of flukes and appeared to be resolving.

Histologically, swollen epithelium protruding at the leading hemibranch edge was hyperplastic, spongiotic, and variably infiltrated with eosinophils and other leukocytes (Figures 2E,F). Hyperplastic tissue had substantially distorted the basal lamina that surrounds the efferent filamental vessels/sinusoids and associated connective tissue. Mucous cells were sporadic in distribution throughout the mucosa of protruding tissue. Mast cells and to a lesser degree lymphocytes, seemingly resident in unaffected filamental peripheries of both parasitized and non-parasitized fish, were less numerous. Immediately adjacent to embedded flukes the lamellae, basal epithelium, basement membrane, and connective tissues of the contacted filaments were replaced by a mass of predominantly intraepithelial eosinophils, lymphocytes, and other mononuclear leucocytes (Figures 2D,G,H). This construct was also vascularized where erythrocytes were visible within capillary lacunae (Figures 2D–G). Lymphocytes dominated in number toward the afferent arterial region of the filament trailing edge or intra-branchial septum (Figure 2D). Eosinophils were easily located in multiple tissue compartments including the filamental epithelium, vascular lumen, connective tissue, diffuse lymphoid tissue, and host–pathogen interstices (Figures 2D,G–I). Eosinophils, often degranulating, were also numerous within interstices between the parasite and gill tissues (Figure 2I). Cells in these regions were often seemingly attached or adherent to the syncytial tegument of embedded *H. thynni* (Figure 2I). In some cases a mixed epithelial/inflammatory layer (of host origin) was also adherent to the parasites tegument. Hemorrhage was infrequently detected although haematin and fibrin was discernible within the digestive apparatus of most flukes. Chloride cells were rarely observed in the trailing afferent filamental region compared to un-parasitized filaments.

#### Pseudocycnus appendiculatus

**Figure 3:**
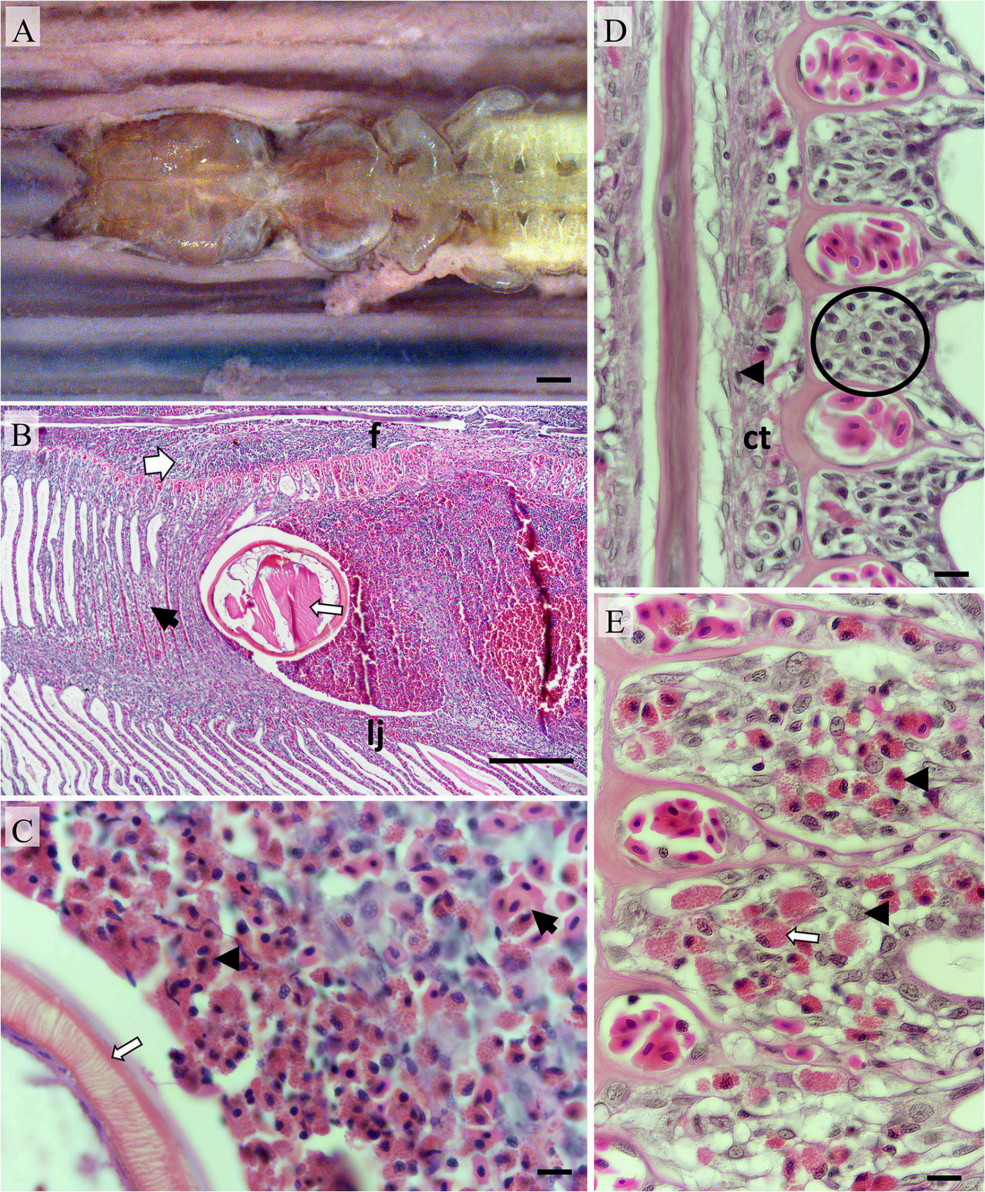
**Pathomorphology of gill filaments parasitized by ***Pseudocycnus appendiculatus***. (A)** The leading edge of a fixed hemibranch showing a *P. appendiculatus* clasping to a filament; Bar = 1 mm. **(B)** Modified antenna (thin arrow) embedded between a filament (f) and lamellae juncture (lj). Inflamed connective tissue (broad arrow) and hyperplastic lamellae (black arrow) are evident; Bar = 1 mm (H&E). **(C)** Eosinophils (black arrowhead) surrounding embedded antenna (white arrow) with haemorrhaging erythrocytes present nearby (black arrow); Bar = 10 μm (H&E). **(D)** Saggitally aligned section showing the basal epithelium (encircled) from a filament distant from the attachment site, Occasional granulocytes (black arrowheads) are present in connective tissue vessels; Bar = 10 μm (H&E). **(E)** Basal epithelium from a filament immediately adjacent to the attachment site (on the same sectional plane) infiltrated with eosinophils (black arrowheads) and putative mast cells (white arrow); Bar = 10 μm (H&E).

Grossly there were no overt signs of swelling or other obvious alterations at the attachment sites of *P. appendiculatus* although minor swelling and hemorrhage was sometimes evident under a dissecting scope. *P. appendiculatus* were generally found attached in the medial to distal portion of filaments where the parasite's modified antennae and maxilliped pair were clamped around the leading edge of the filament (Figure 3A).

Histologically, localized inflammation, hemorrhage, and hyperplasia were most marked in the immediate vicinity to the parasite's clamping apparatus (Figures 3B,C). Eosinophils infiltrated the interface between host tissue and copepod surfaces frequently in substantial numbers (Figure 3C). Numerous eosinophils and occasional mast cells had infiltrated the basal epithelium of the filaments (Figures 3D,E). Epithelial hyperplasia was noted upon the leading filamental edges and these regions were variably infiltrated with eosinophils. Hyperplastic epithelium distally fused neighboring lamellae to form vesicles and/or crypts containing degraded erythrocytes and leucocytes predominantly eosinophils and macrophages. Mucus cells were more numerous upon lamellae adjacent to the attachment site in comparison to those distant. The trailing filamental regions were not penetrated by grasping antennae and hence appeared markedly less affected. Chloride cell distributions in these regions were similar to adjacent un-parasitized regions.

#### Euryphorus brachypterus

**Figure 4:**
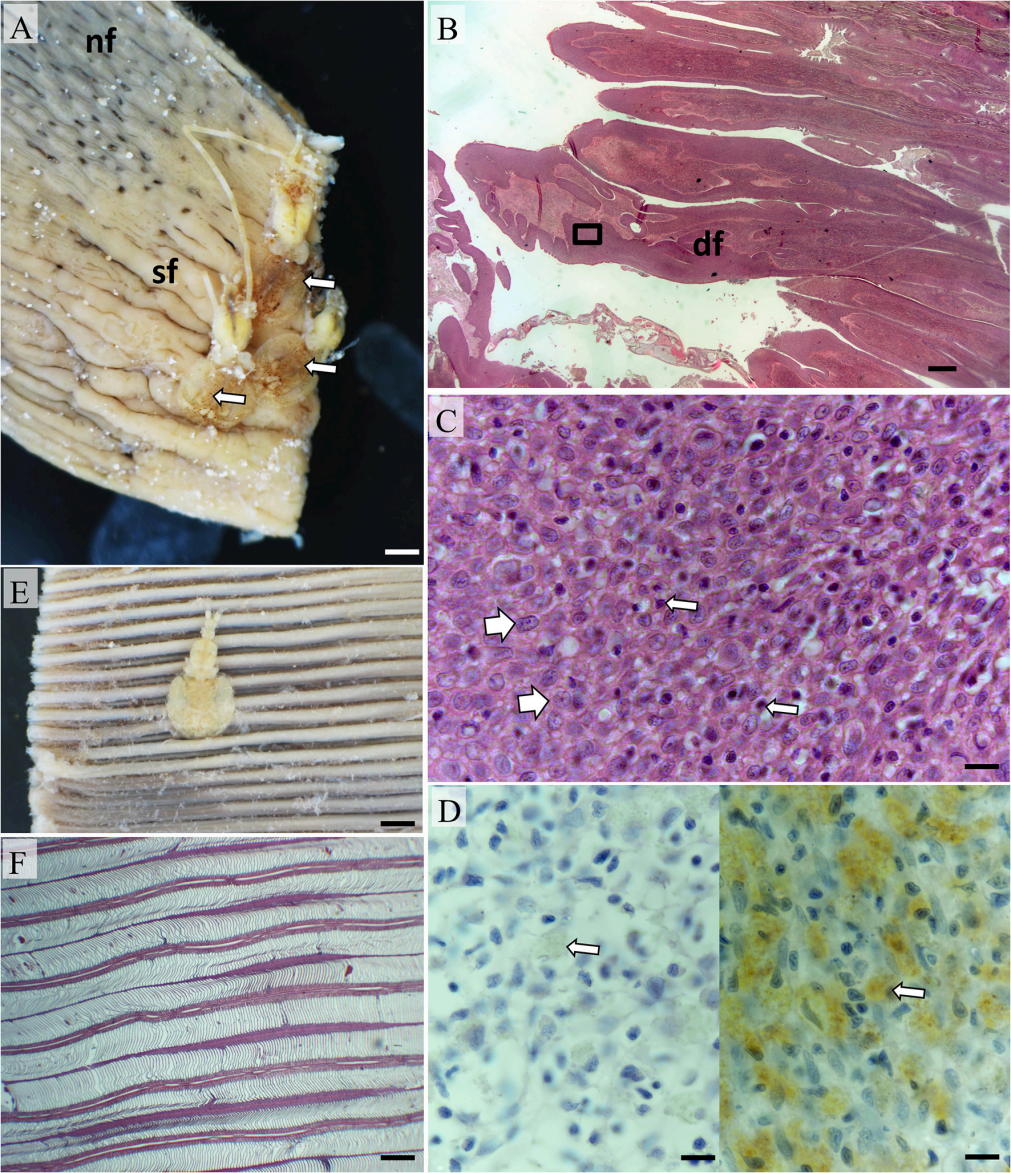
**Pathomorphology of gill filaments parasitized by ***Euryphorus brachypterus***. (A)** Three *E. brachypterus* copepods attached basally on a fixed hemibranch (white arrows). Note the swollen filaments adjacent and extending distally (sf) in comparison to distant filaments with normal sub-gross appearance (nf); Bar = 5 mm. **(B)** Histological section from **(A)** showing distortion of filaments (df); Bar = 1 mm (H&E). **(C)** High magnification of box in **(B)** showing epithelial hyperplasia (white arrows) with occasional small mononuclear cells present (thin arrows). The surfaces of swollen filaments were rich in mucus cells (data not shown); Bar = 10 μm (H&E). **(D)** Putative mast cells (right panel—white arrow) were also numerous but sporadically distributed in the leading edge of affected filaments (left panel shows isotype control); Bar = 10 μm (H&E). **(E)** *E. brachypterus* copepod attached distally on a fixed hemibranch, sub-grossly there is little evidence of superficial tissue reactivity; Bar = 5 mm. **(F)** A section cut 1 mm immediately below the parasite in **(E)** likewise showing little tissue reactivity—note normal appearance of lamellae; Bar = 2 mm (H&E).

Gross changes associated with the attachment of *E. brachypterus* varied with proximity to the gill arch. Where *E. brachypterus* had attached distally or medially upon filaments swelling and hemorrhage was limited. However, where attachment was proximal to the arch pronounced swelling was evident (Figure 4A) particularly where multiple, abutting parasites were present.

Histological examination demonstrated variable host tissue responses relative to the filament attachment location of *E. brachypterus*. Where *E. brachypterus* were located proximal to the arch, more pronounced structural changes to filaments were observed superficially (Figures 4A,B). In addition to mucosal hyperplasia some filaments were distorted by hyperplastic epithelial, chondroid, and fibroblastic cells although the former dominated (Figure 4C). These transformations were also evident in the lamellar regions in some cases. Sloughed epithelium, cellular debris, PAS positive materials, fibrin, and erythrocytes were also prominent (data not shown). Mast cells were sporadic in distribution although substantial concentrations were evident (Figure 4D) although it was unclear whether this was artifactual due to distortion of filaments. Distal lamellar fusion due to a focal epithelial hyperplasia was observed and occasionally associated with inter-filamental vesicles or crypts. Hyperplastic epithelial tissue and resultant vesicles were infiltrated with leucocytes although eosinophils were only occasionally noted in contrast to the aforementioned parasites. In distal to medial locales, mild mucosal hyperplasia of the filament leading edge was evident immediately beneath attached parasites on the leading filament edge. However, the lamellar and trailing filamental regions were unaffected by any substantial morphological alteration (Figures 4E,F).

## Discussion

This study has presented initial descriptions of the branchial pathomorphological response of *Thunnus macoyii* to infection with the gill fluke *H. thynii* and two copepodan parasites *P. appendiculatus* and *E. brachypterus. H. thynni* infections were the most pronounced in terms of tissue remodeling and structural distension due to hyperplastic (potentially metaplastic) and inflammatory processes. These responses may bedriven by the stationary life style of this parasite. It had been previously postulated that swelling caused by the parasite may improve its attachment to SBT gills as suggested for other closely related parasites and hosts (Dawes, 1940; Deveney et al., 2005; Hayward, 2005). Indeed, mammalian helminth infections are sometimes able to be maintained due to the parasite's ability to modulate or resist innate host responses (MacDonald et al., 2002; Anthony et al., 2007). However, given the gross observations of dead flukes and histological evidence of tegument damage observed here; tissue remodeling and inflammation may ultimately result in the eventual expulsion of the fluke from SBT gills analogous to helminth destruction in other organisms (Shin et al., 2009; Makepeace et al., 2012).

Hyperplasia of gill epithelium is a common response to parasitic infections and was also observed in the areas of attachment of *P. appendiculatus* and *E. brachypterus*, although not to the same extent as with *H. thynni*. This may be because copepods are relatively mobile and may change their attachment site (Sutherland and Wittrock, 1985) and do not penetrate or feed in the same manner as monogeneans. Congruently, reactivity is likely to be influenced by the antigenic profile of the parasites particularly flukes that are able to generate a substantive inflammatory tissue response (Klion and Nutman, 2004).

Inflammatory infiltrates were dominated by putative eosinophils with morphology consistent with those described from other species of fish (Roberts and Ellis, 2013). They are the most common granulocytes in the peripheral circulation of wild SBT and yellowfin tuna (Hine, 1992; Rough et al., 2005). Eosinophils were scant in most gill tissue compartments of unparasitized tuna. Contrastingly, substantially larger numbers were present in nearly all tissue compartments in the gills infected by *H. thynni* and *P. appendiculatus*. Eosinophil presence in host-pathogen interstices was prominent and degranulation was consistently observed. In zebrafish, *Brachydanio rerio* (Hamilton, 1822), eosinophils expressed genes are important for the activities of mammalian eosinophils and increased in numbers in the intestine in response to infection with *Pseudocapillaria tomentose* (Dujardin, 1843), a parasitic nematode (Balla et al., 2010). Zebrafish eosinophils degranulated when exposed to the nematode *Heligmosomoides polygyrus* (Dujardin, 1845) extract *in vitro* (Balla et al., 2010) and direct cytotoxic destruction of helminths has been reported in mammalian studies (Klion and Nutman, 2004) This suggests that fish eosinophils share common responses to helminth infections as with other vertebrates. Functionally, eosinophils are granulocytes involved in innate immune surveillance, including assistance with T lymphocyte-mediated humoral immune responses, and tissue remodeling (Hogan et al., 2008). When gill filaments were parasitized with *H. thynni*, the parenchyma and connective tissues of the central and trailing regions were transformed or displaced by a proliferative mass of capillarized lymphoid tissue (infiltrated with eosinophils but not mast cells). Interestingly, underlying the mucosa of the leading edge and trailing edges of non-parasitized filaments, intra-epithelial cells morphologically consistent with lymphocytes along with putative mast cells were consistently observed. The study of gill associated lymphoid tissues is an emerging field although it is suggested that teleosts possess a diffusely arranged mucosa associated lymphoid tissue (MALT) (Salinas, 2015). Lymphocyte infiltration is an inflammatory reaction common to a range of gill infections (Roberts and Ellis, 2013), however to the best of our knowledge, a transformative lymphoid proliferation of the type described here has not been previously reported in teleost gills. Further anatomical and functional studies of mucosa associated lymphoid tissue in the gills of this species would be fundamentally beneficial to further our understanding in this field. In this study, differentiation between eosinophils and larger granulocytes with packed finer granules was achieved by exploiting tryptase antibody cross reactivity, non-reactivity to *Na/K/ATPase* antibody and the basic protein staining properties of eosinophils at high pH. Granulocytes described as eosinophilic granular cells were observed in SBT infected with the blood fluke *Cardicola forsteri* (Poche, 1926) (Colquitt et al., 2001) although no detailed description was presented. Mast cells from Atlantic bluefin tuna *Thunnus thynnus* were observed in semi-thin sections in filaments parasitized by *P. appendiculatus* (Pleić et al., 2015). Mast cells are a multi-mechanistic principle inflammatory cell participating in a range of response scenarios (Lee et al., 1986; Amin, 2012; Urb and Sheppard, 2012). Mast cells contain various mediators such as histamine, heparin, tryptase, serotonin as well as antimicrobial peptides, lysosomal enzymes, eicosanoids, cytokines, and reactive oxygen species (Gilfillan et al., 2011). Across the infraclass teleostea, there is a significant amount of variation in these constituents between families, genera, and species which is further complicated by different approaches and combinations of fixation, labeling, and staining (Reite and Evensen, 2006). In mice, mast cells were initially classed as either mucosal or connective tissue mast cells on the basis of differential fixation and staining properties of their secretory granules (Enerback, 1966). It is not known whether a similar delineation occurs in teleosts. Definitive characterization of mast cells in SBT gills requires further exploration.

Chondroid, fibroblastic and epithelial hyperplasia were seen in gills of some SBT infected with *E. brachypterus*. Severe epithelial hyperplasia, fibroplasia, and inflammation leading to formation of nodules were observed in the gills of coral trout, *Plectropomus leopardus* (Lacepède, 1802) infected with high numbers of the siphonostome copepod *Dissonus manteri* (Kabata, 1966; Bennett and Bennett, 2001).

Differing parasite feeding strategies may result in contrasting pathology. While *H. thynni* and *P. appendiculatus* feed on host blood (Halton and Jennings, 1965; Kabata, 1966), *E. brachypterus* feeds on host mucus. The host response was milder in *P. appendiculatus* infection than in *H. thynni*, however there were some similarities, including hyperplastic and inflammatory responses, involving eosinophils at the site of attachment. While most of the cellular responses observed here were consistent with published information on similar species of parasites (Natarajan and Nair, 1972; Mansell et al., 2005) no hemorrhage was seen in *E. brachypterus* infection as reported previously in Atlantic bluefin tuna (Williams and Bunkley-Williams, 1996). This may be due to the low intensity of infection in our study, the hemorrhage described in Atlantic bluefin tuna was due to infection with a very high number of these parasites (Williams and Bunkley-Williams, 1996). This study investigated the effects of the presence of the adult parasites on the gills of SBT. Other life stages could cause different pathology as was shown for *P. leopardus* and the siphonostome copepod *D. manteri* (Bennett and Bennett, 2001). The host response observed here at the cellular level was focal and suggests that the fish are able to mount an effective response under culture conditions. This supports the infection patterns observed for these parasites, in particular the decline in their numbers during ranching of SBT (Hayward et al., 2007, 2008; Kirchhoff et al., 2011). The low impact at both the cellular and organism level could be due to a relatively low intensity of infection observed for these parasites (Hayward et al., 2007, 2008; Kirchhoff et al., 2011).

On the basis of the host response reported here and the low intensity of infection observed in other studies these gill ectoparasites are currently considered a low risk for adult wild and ranched SBT.

## Author contributions

MA made significant contributions to design, data acquisition, analysis, and interpretation of data. Drafted the work and revised it critically for important intellectual content. Gives final approval for the version to be published and agrees to be accountable for all aspects of the work in ensuring that questions related to the accuracy or integrity of any part of the work are appropriately investigated and resolved. CH and BN made substantial contributions to the conception and design of the work. Critically revised for important intellectual content. Gives final approval for the version to be published and agrees to be accountable for all aspects of the work in ensuring that questions related to the accuracy or integrity of any part of the work are appropriately investigated and resolved.

### Conflict of interest statement

The authors declare that the research was conducted in the absence of any commercial or financial relationships that could be construed as a potential conflict of interest. The reviewer AG and handling Editor declared their shared affiliation, and the handling Editor states that the process nevertheless met the standards of a fair and objective review.
